# Adaptation of key bacterial vaginosis-associated bacteria to a medium simulating genital tract secretions: a transcriptomic analysis

**DOI:** 10.3389/fgene.2025.1552307

**Published:** 2025-03-26

**Authors:** Lúcia G. V. Sousa, Angela França, Vânia Pinheiro, Christina A. Muzny, Nuno Cerca

**Affiliations:** ^1^ Centre of Biological Engineering (CEB), Laboratory of Research in Biofilms Rosário Oliveira (LIBRO), University of Minho, Braga, Portugal; ^2^ LABBELS – Associate Laboratory, Braga, Portugal; ^3^ Division of Infectious Diseases, University of Alabama at Birmingham, Birmingham, AL, United States

**Keywords:** bacterial vaginosis, polymicrobial biofilms, species interactions, RNA-sequencing, gene expression

## 1 Introduction

Approximately 30% of reproductive-age women around the world are affected by bacterial vaginosis (BV), which is the most common vaginal infection and the main cause of vaginal discharge (Peebles et al., 2019). BV is a polymicrobial vaginal dysbiosis with substantial data supporting the role of sexual transmission of BV-associated bacteria (BVAB) (Fethers et al., 2008; Muzny et al., 2022). In a case of BV, several strict and facultative anaerobic bacteria are increased in number in the vaginal microbiota replacing protective *Lactobacillus* species, which are the major colonizers present in a healthy vaginal microbiota (Rosca et al., 2020b; Chen et al., 2021). These anaerobic species are recognized to be living in a polymicrobial biofilm adhered to vaginal epithelial cells creating the characteristic clue cells (Swidsinski et al., 2024). Despite the identification of multiple bacterial species in cases of BV (Ceccarani et al., 2019), some may have a greater impact on BV development than others (Gilbert et al., 2019; Randis and Ratner, 2019; Castro et al., 2021). Previously, *Gardnerella vaginalis*, *Fannyhessea vaginae*, and *Prevotella bivia* were detected, *in vivo* specimens from women with BV, early in the development of BV (Muzny et al., 2018), and further *in vitro* experiments showed important synergisms between these species suggesting an essential role in the initial stages of BV biofilm formation (Castro et al., 2021). Having this in mind, we recently investigated how these three bacterial species interact when growing in a nutrient-rich medium, which allowed for polymicrobial biofilm growth. Notably, we observed that hundreds of genes were differentially expressed when comparing triple-species with single-species biofilms (Sousa et al., 2024).

However, the adaptation of bacterial species to different growth media results in alterations in the bacteria transcriptome and consequent variations in gene expression (Blair et al., 2013; Smith et al., 2018). As such, the consideration of which culture medium is most suitable to grow a specific species is crucial depending on the experiments that will be performed and the factors that need to be analyzed. Although the use of a nutrient-rich culture medium allows a better growth of bacterial species in laboratory settings, especially when growing fastidious microorganisms, using culture media that mimic *in vivo* conditions is preferable when trying to evaluate how microorganisms behave *in vivo*-like conditions (Pouget et al., 2022). For the study of BV it is useful to have a medium that simulates the vaginal environment, and the medium simulating genital tract secretions (mGTS) contains several amino acids, as well as albumin, urea, and mucin found in the vaginal fluid (Tietz and Klein, 2018).

Thus, to further investigate how the vaginal environment affects the transcriptome of the BV polymicrobial biofilm, we aimed to explore how transcriptome adaptations observed in the nutrient-rich medium would compare with adaptations to mGTS, which partially simulates *in vivo* vaginal conditions.

## 2 Materials and methods

### 2.1 Bacterial strains and growth conditions

G. *vaginalis* ATCC 14018, *F*. *vaginae* ATCC BAA-55, and *P*. *bivia* ATCC 29303 were used in this study. The bacteria were kept in Brain Heart Infusion medium (Liofilchem, Roseto degli Abruzzi, Italy) supplemented with 23% of glycerol (98%, Panreac, Darmstadt, Germany) at −80°C. Before each experiment, they were grown on Columbia Base Agar (Liofilchem) plates supplemented with 5% of defibrinated horse blood (CBA) (Thermo Fisher Scientific, Lenexa, KS) for 48 h at 37°C and in anaerobic conditions (Anaerocult™ A, Merck Millipore, Taufkirchen, Germany). New York City III (NYCIII) medium, a nutrient rich medium used for the growth of fastidious microorganisms, was prepared as previously described (Rosca et al., 2020a). mGTS medium, which simulates vaginal tract secretions, was adapted from previous studies (Liu et al., 2011; Stingley et al., 2014), and was prepared for the purpose of this study as previously described (Sousa et al., 2025).

### 2.2 Biofilm formation

Triple-species biofilms of *G. vaginalis*, *F. vaginae*, and *P. bivia* were formed on 24-well culture plates (Orange Scientific, Braine-l’Alleud, Belgium), in either NYCIII medium or mGTS, using the competitive model previously described (Rosca et al., 2022a). The biofilm competitive model considers that all species are present on the initiation of BV biofilm, and thus the species are incubated at the same time in the plates to form biofilm. For the biofilms formed in NYCIII medium, a suspension of each species was prepared and incubated at 37°C in anaerobic conditions for 24 h. For the biofilms grown in mGTS, since this species have limited growth in planktonic cultures in this medium, a suspension of each species was prepared from bacterial biomass growing on CBA plates on the same day of biofilm formation. Thereafter, for both protocols, the bacterial concentration was adjusted to 9 × 10^7^ CFU/mL by reading the optical density at 620 nm (Castro et al., 2021). The bacterial suspensions were then dispensed on the wells of the plate for a total volume of 1 mL, with a final concentration of 1 × 10^7^ CFU/mL for each species, and incubated for 48 h at 37°C under anaerobic conditions. For the characterization of the composition of triple-species biofilms by qPCR, the biofilm medium was removed and the biofilms washed once with 0.9% NaCl (Sigma, Germany), followed by mechanical detachment from the plates in 1 mL of 0.9% NaCl. The content of the wells was subsequently combined. Afterwards, genomic DNA was extracted and qPCR quantification was performed using specific primers for each bacterial species, as previously described (Rosca et al., 2022b). Using calibration curves previously designed (Lameira et al., 2024), the concentration of each species in the triple-species biofilm was determined. For RNA-seq experiments, the biofilm medium was removed, the biofilms were washed once with 1 × phosphate-buffered saline (PBS) (Thermo Fisher Scientific) and then suspended in RNA protect Bacteria Reagent (Qiagen, MD, United States), diluted 2:1 in 1 × PBS. The samples were then centrifuged at 5,000 *g* for 10 min at room temperature. These experiments were repeated at least three times.

### 2.3 RNA extraction

Twelve triple-species biofilms formed in either NYCIII or mGTS were pooled to obtain samples with enough RNA concentration for further analysis. RNA extraction was performed using the RNeasy Mini Kit (Qiagen), according to the manufacturer’s instructions, with some minor modifications (França et al., 2012). First, cells were suspended in 600 μL of lysis buffer RLT and the volume was transferred to a tube with 0.1 mm zirconium beads (Merck, Darmstadt, Germany). Cells were lysed using the BeadBug 6 Microtube Homogenizer (Benchmark Scientific, NJ, United States) at maximum speed for 35 s. The lysis cycle was repeated four times and the samples were kept on ice for 5 min between cycles. Then, the samples were centrifuged, and the supernatant was recovered into a new tube. Ethanol at 70% (Thermo Fisher Scientific) was added in the same proportion (vol:vol) to the supernatant and the solution was transferred into an RNeasy Mini spin column. After the washing steps, total RNA was eluted in RNase-free water (Grisp) and the samples were treated with Turbo DNase (Invitrogen, Waltham, Massachusetts, United States) to degrade genomic DNA following the manufacturer’s instructions for rigorous protocols.

### 2.4 cDNA library preparation and sequencing

RNA quality was assessed using the Agilent 2100 Bioanalyzer (Agilent, CA, United States) and only samples with RNA quality indicators above seven were used. RNA-seq libraries were prepared using Lexogen’s CORALL™ Total RNA-seq kit (Lexogen, Vienna, Austria) with 100 ng of total RNA from each sample. Before RNA-seq, rRNA was removed using the RiboCop for Bacteria (Mixed Bacterial Samples META) rRNA Depletion kit (Lexogen). Sequencing libraries were evaluated for quality on a Fragment Analyzer System (Agilent) and quantified with Qubit™ dsDNA HS Assay Kit (Invitrogen). Sequencing data were generated using Illumina NextSeq 2000 Sequencing from single-end reads (SR100). FastQ files were generated *via* Illumina bcl2fastq2 (v.2.17.1.14). The quality of individual sequences was evaluated using FastQC software after adapter trimming with cutadapt software (1.18).

### 2.5 RNA-sequencing data analysis

FastQ files were then analyzed using CLC Genomics Workbench software (Qiagen, version 21.99). Quality trimming, including both quality scores and nucleotide ambiguity, was performed using the CLC genomics workbench default settings (Supplementary Table S1). Alignment of each species’ sequences was performed using *G. vaginalis* NCTC10287 (NCBI reference sequence: NZ_LR134385.1), *F. vaginae* FDAARGOS_934 (NCBI reference sequence: CP065631.1), and *P. bivia* DSM 20514 (NCBI reference sequence: NZ_JH660658.1; NZ_JH660659.1; NZ_JH660660.1) as reference genomes, also using the software default settings (Supplementary Table S2). Differential expression analysis was performed using Reads Per Kilobase per Million (RPKM) mapped fragments as the normalization strategy with triple-species biofilms grown in NYCIII as controls. Baggerley’s test was applied to identify statistically significant alterations. Fold changes > 2 or < −2 and with a false discovery rate (FDR) *p-*value < 0.05 were considered significant and used for further bioinformatics analyses. Raw and analyzed datasets were deposited in the Gene Expression Omnibus database under the reference GSE279623.

### 2.6 Functional annotation

Functional enrichment of differentially expressed genes was assessed using the Search Tool for the Retrieval of Interacting Genes/Proteins (STRING, version 11.5) based on Gene Ontology (GO) and Kyoto Encyclopedia of Genes and Genomes (KEGG) databases. Classes with FDR-adjusted *p*-values < 0.05 were considered for enrichment. REVIGO (version 1.8.1) was used for removing redundant GO terms. UniProt was used to find the homology of hypothetical proteins.

### 2.7 Statistical analysis

Principal Component Analysis (PCA) graphs and heatmaps were created using the CLC Genomics Workbench (version 21.99). All other figures and analyses were performed using GraphPad Prism version 8.2 (La Jolla, CA, United States of America). Statistical analysis was performed using two-way ANOVA with Tukey’s multiple comparisons test. Statistical differences were considered when *p* < 0.05.

## 3 Data description

To study the influence of simulated *in vivo* conditions on the interactions of key BVAB, we analyzed the transcriptome of a triple-species biofilm composed of *G. vaginalis*, *F. vaginae,* and *P. bivia*, when biofilms were grown in a rich medium versus a medium mimicking genital tract secretions.

Triple-species biofilms formed in mGTS were also analyzed by qPCR to determine each species’ percentage, revealing that the great majority of the biofilm was composed of *G. vaginalis*, followed by *P. bivia*, and *F. vaginae*, as shown in Supplementary Figure S1.

Regarding RNA-seq data, the quality of the sequencing data was assessed by evaluating some of the summary parameters from the trimming and mapping steps as described at Supplementary Tables S3, S4, respectively. The number of sequencing reads ranged between 13137570 and 20716698. The results of mapping to the reference genome sequence revealed a percentage of mapping between 1.82% and 75.76% for the NYCIII medium and between 0.63% and 67.48% for the mGTS medium. Overall, the sequences from *P. bivia* were the ones with the lowest percentages of mapping.

The PCA plots, depicted in Supplementary Figures S2–S6, revealed differences among the triplicates analyzed for each condition. Triplicates of biofilms formed in mGTS were more similar than those formed in NYCIII medium for *G. vaginalis* and *P. bivia*. In contrast, more differences were found among the biofilm triplicates in the mGTS condition for *F. vaginae*. These variations are likely a result from biological heterogeneity found in biofilms (Sousa et al., 2014). However, a clear separation from the triplicates in NYCIII and mGTS was observed for all the species, indicating that the two conditions caused alterations in the transcriptome of the three species.

**FIGURE 1:**
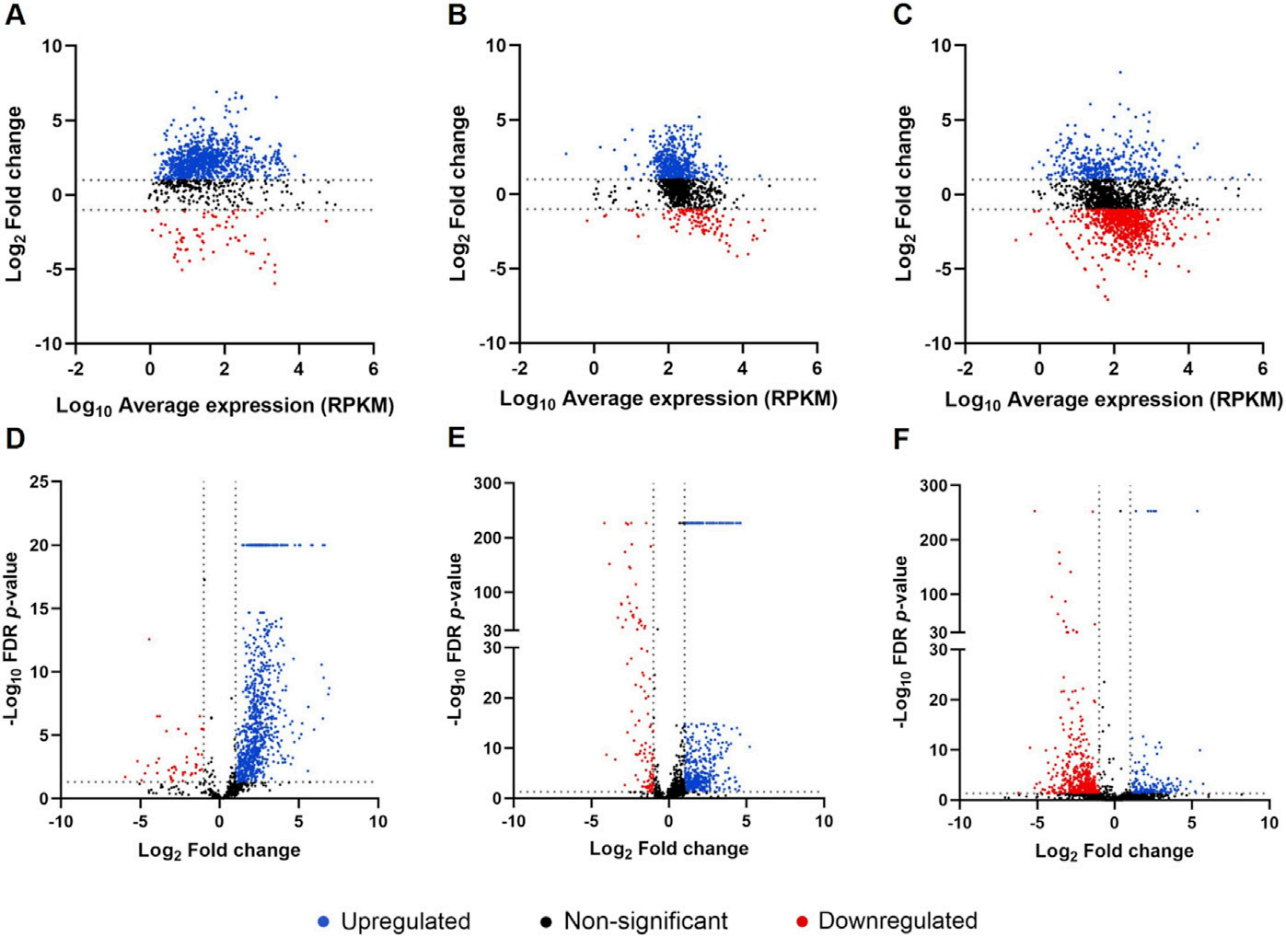
Analysis of differentially expressed genes in triple-species biofilms in mGTS Distribution of differentially expressed genes by MA plots for *Gardnerella vaginalis* **(A)**, *Fannyhessea vaginae* **(B)**, and *Prevotella bivia* **(C)** and by volcano plots for *G. vaginalis* **(D)**, *F. vaginae* **(E)**, and *P. bivia* **(F)**. Each point represents a gene, blue points represent upregulated genes, red points represent downregulated genes, and black points represent genes with non-significant differential expression (significant differential expression was considered for genes with fold change > 2 or < −2 and FDR *p*-value < 0.05). Graphics were plotted using GraphPad Prism.

The distribution of gene expression was evaluated in both conditions for each species, as shown in Supplementary Figure S7. The box plots, which display the distribution of the RPKM values, revealed a similar distribution of the data in NYCIII and mGTS for the three species. The scatter plots, which represent the correlation between RPKM values in the NYCIII and mGTS conditions, showed that more genes were upregulated in the mGTS condition for *G. vaginalis* and *F. vaginae* (more genes displayed above the line), whereas for *P. bivia* more genes were downregulated in the same condition (more genes displayed below the line). Finally, heat maps depicting the normalized gene expression pattern in the three species across conditions and triplicates showed a clear alteration in gene expression from NYCIII to mGTS condition (Supplementary Figures S8–S12). For differential gene expression analysis, biofilms formed in the NYCIII medium were used as the control. A total of 1,315, 1,202, and 2,184 differentially expressed genes were found for *G. vaginalis*, *F. vaginae*, and *P. bivia*, respectively. MA and volcano plots (Figure 1) showed that the expression of the majority of the genes has a fold change between −32 and 32 (−5 < Log_2_ > 5). Moreover, for *F. vaginae* and *P. bivia,* a wide range of FDR *p*-values was found in comparison with *G. vaginalis*.

For further analysis, only the genes whose fold change values were > 2 or < −2 and with FDR *p*-value < 0.05 were considered. This analysis resulted in 927, 492, and 166 upregulated genes for *G. vaginalis*, *F. vaginae*, and *P. bivia,* respectively. Moreover, 37, 104, and 490 genes were found to be downregulated in biofilms formed in mGTS for *G. vaginalis*, *F. vaginae*, and *P. bivia*, respectively. The top ten most upregulated and downregulated genes for each species and their respective functions are detailed in Table 1.

**TABLE 1:** List of top 10 genes most upregulated and downregulated in *Gardnerella vaginalis*, *Fannyhessea vaginae* and *Prevotella bivia*.

Gene	Description	Fold change	FDR *p*-value
*G. vaginalis* upregulated genes			
EL180_RS02015	Anchored repeat-type ABC transporter permease subunit	120.52	1.99 × 10^−9^
EL180_RS02030	Choice-of-anchor M domain-containing protein	115.23	5.89 × 10^−9^
EL180_RS05235	ABC transporter permease	98.02	0.00
EL180_RS05230	ABC transporter substrate-binding protein	94.13	2.92 × 10^−10^
EL180_RS02035	Choice-of-anchor M domain-containing protein	91.12	4.83 × 10^−7^
EL180_RS05240	ABC transporter permease	90.80	0.00
EL180_RS02025	Anchored repeat ABC transporter, substrate-binding protein	85.80	2.63 × 10^−11^
EL180_RS02020	Anchored repeat-type ABC transporter ATP-binding subunit	61.94	3.69 × 10^−6^
EL180_RS02430	Hypothetical protein	58.14	0.00
EL180_RS05245	ABC transporter ATP-binding protein	54.61	0.00
*G. vaginalis* downregulated genes			
EL180_RS06735	Prevent-host-death protein	−61.95	2.02 × 10^−2^
*pstC*	Phosphate ABC transporter permease subunit PstC	−36.29	1.10 × 10^−3^
EL180_RS06740	Hypothetical protein	−30.34	3.62 × 10^−2^
EL180_RS05315	Type II toxin-antitoxin system RelB/DinJ family antitoxin	−26.11	1.01 × 10^−2^
EL180_RS05320	Type II toxin-antitoxin system RelE/ParE family toxin	−22.81	2.54 × 10^−3^
*chvE*	Sugar ABC transporter substrate-binding protein	−15.79	1.47 × 10^−3^
EL180_RS07255	Hypothetical protein	−15.32	3.22 × 10^−7^
EL180_RS06515	Amino acid ABC transporter ATP-binding protein	−15.30	9.51 × 10^−3^
EL180_RS05440	Type II toxin-antitoxin system RelB/DinJ family antitoxin	−14.33	6.89 × 10^−4^
EL180_RS05410	GNAT family N-acetyltransferase	−13.71	3.22 × 10^−7^
*F. vaginae* upregulated genes			
I6G91_04405	Two-component sensor histidine kinase	36.71	5.28 × 10^−11^
I6G91_04285	MerR family transcriptional regulator	24.36	0.00
I6G91_00035	NCS2 family permease	24.09	1.97 × 10^−13^
I6G91_05015	ABC transporter ATP-binding protein	24.08	0.00
I6G91_05010	Sensor histidine kinase	23.64	0.00
I6G91_05125	Phosphoketolase	23.56	0.00
I6G91_04360	Hypothetical protein	23.35	3.75 × 10^−3^
I6G91_04355	Plasmid mobilization relaxosome protein MobC	22.57	2.62 × 10^−2^
I6G91_02215	DMT family transporter	21.56	0.00
I6G91_03530	FIVAR domain-containing protein	20.78	9.31 × 10^−15^
*F. vaginae* downregulated genes			
*grcA*	Autonomous glycyl radical cofactor GrcA	−17.96	0.00
I6G91_02675	DNA starvation/stationary phase protection protein	−16.27	2.23 × 10^−9^
*pflB*	Formate C-acetyltransferase	−14.31	1.35 × 10^−152^
*raiA*	Ribosome-associated translation inhibitor RaiA	−11.04	1.89 × 10^−8^
I6G91_01400	Extracellular solute-binding protein	−9.80	3.53 × 10^−54^
I6G91_03480	Spx/MgsR family RNA polymerase-binding regulatory protein	−8.45	4.50 × 10^−80^
I6G91_00320	Co-chaperone GroES	−8.37	1.23 × 10^−78^
*trxA*	Thioredoxin	−7.10	5.83 × 10^−175^
*groL*	Chaperonin GroEL	−6.90	3.21 × 10^−50^
I6G91_03595	Class II fructose-bisphosphate aldolase	−6.80	0.00
*P. bivia* upregulated genes			
*nrdG*	Anaerobic ribonucleoside-triphosphate reductase activating protein	52.41	5.95 × 10^−4^
*crcB*	Fluoride efflux transporter CrcB	45.33	1.14 × 10^−10^
PREBIDRAFT_RS02020	Thiamine phosphate synthase	40.36	0.00
PREBIDRAFT_RS00910	YitT family protein	36.86	1.94 × 10^−2^
PREBIDRAFT_RS02035	Thiamine phosphate synthase	36.73	2.21 × 10^−2^
*thiD*	Bifunctional hydroxymethylpyrimidine kinase/phosphomethylpyrimidine kinase	29.66	2.10 × 10^−4^
PREBIDRAFT_RS08375	Replication initiation protein	24.67	5.45 × 10^−3^
PREBIDRAFT_RS00825	Hypothetical protein	18.93	2.63 × 10^−4^
PREBIDRAFT_RS00830	Site-specific integrase	18.89	1.05 × 10^−3^
PREBIDRAFT_RS08215	Excisionase family DNA-binding protein	16.39	1.90 × 10^−3^
*P. bivia* downregulated genes			
PREBIDRAFT_RS11775	Hypothetical protein	−74.39	4.03 × 10^−2^
PREBIDRAFT_RS07240	4Fe-4S binding protein	−44.31	3.50 × 10^−11^
PREBIDRAFT_RS06785	Aspartate ammonia-lyase	−35.96	0.00
PREBIDRAFT_RS00195	Desulfoferrodoxin family protein	−35.81	4.01 × 10^−3^
PREBIDRAFT_RS03760	IS982 family transposase	−32.90	6.15 × 10^−4^
PREBIDRAFT_RS00200	Hypothetical protein	−28.38	3.32 × 10^−4^
*nadD*	Nicotinate (nicotinamide) nucleotide adenylyltransferase	−27.14	3.26 × 10^−2^
*cysK*	Cysteine synthase A	−25.28	7.08 × 10^−3^
*hcp*	Hydroxylamine reductase	−22.78	9.51 × 10^−3^
PREBIDRAFT_RS11515	Hypothetical protein	−21.63	1.18 × 10^−10^

To complete the analysis, a GO enrichment analysis was performed for the upregulated and downregulated genes of the three species and the results are reported in Supplementary Figures S13-S15. The main categories of GO including biological processes, molecular functions, and cellular components were considered. For *G. vaginalis*, most of the upregulated genes were found associated with biological processes, mainly cellular and metabolic processes. Among the downregulated genes, enrichment was only found for molecular functions, namely, for the active transmembrane transporter activity. For *F. vaginae*, more enrichment terms were found in the cellular components category, followed by molecular functions and biological process. The cellular anatomical entity and cellular process were the terms with the highest percentage of genes identified for both upregulated and downregulated genes, however, more terms of enrichment were found among the upregulated genes. For *P. bivia*, more GO terms were found for the category of biological process, followed by molecular functions and cellular components. GO enrichment was mostly observed within the downregulated genes which were mainly associated with metabolic processes. The upregulated genes were mainly associated with transport activity. The highest percentage of genes was observed for the term cellular anatomical entity for both upregulated and downregulated genes.

Regarding KEGG pathways analysis (Supplementary Figure S16), different pathways were found enriched within the upregulated and downregulated genes for each of the three species. KEGG pathways among upregulated genes were only found for *G. vaginalis* with the identification of metabolic pathways, while for downregulated genes ABC transporters were identified. For *F. vaginae* and *P. bivia*, KEGG pathways were only found enriched within the downregulated genes and were mainly associated with metabolic pathways.

Overall, this work highlights transcriptomic alterations in three key BVAB when growing *in vivo*-simulating conditions compared to a nutrient-rich growth medium. It is important to note that, while these differences may be influenced by the growth medium, they may also reflect the shift in species abundance within the biofilm. Additionally, since the growth of some species, particularly *F. vaginae*, is very limited in the mGTS medium, the observed transcriptomic alterations for this species may result from stress responses caused by the lack of capacity to survive in this medium, rather than a response to the functional adaptation to the medium conditions. Further investigation is needed to elucidate the mechanisms involved in the transcriptomic alterations in these conditions and the contributions to the development of incident BV, including the specific interactions between key bacterial species.

## Data Availability

The datasets presented in this study can be found in online repositories. The names of the repository/repositories and accession number(s) can be found in the article/Supplementary Material.
