# Supplementary material for: Adaptation of key bacterial vaginosis-associated bacteria to a medium simulating genital tract secretions: a transcriptomic analysis
Source: Front Genet. 2025 Mar 26;16:1552307. doi: 10.3389/fgene.2025.1552307 (PMC11979175; doi:10.3389/fgene.2025.1552307)
Supplement: Supplementary file 1 [file DataSheet1.docx]

Supplementary Material

**Supplementary Table 1.** Trimming parameters applied for the analysis of raw RNA-seq reads using CLC Genomics Workbench (version 21.99)

| **Quality trimming** | |
| --- | --- |
| Quality limit (Phred scale) | 0.05 |
| **Trim ambiguous nucleotides** | |
| Maximum number of ambiguities allowed | 2 |

**Supplementary Table 2.** Mapping parameters applied for the analysis of raw RNA-seq reads using CLC Genomics Workbench (version 21.99)

| **Parameter** | |
| --- | --- |
| Mismatches | 2 |
| Insertion cost | 3 |
| Deletion cost | 3 |
| Length fraction | 0.8 |
| Similarity fraction | 0.8 |
| Maximum number of hits for a read | 10 |
| Strands specificity | both |

**Supplementary Table 3.** Quality trimming summary for triple-species biofilms sequences in NYCIII and mGTS. For each condition triplicates of triple-species biofilms were analyzed

| **Condition** | **Replicates** | **Nr of reads** | **Avg. Length** | **Nr of reads after trim** | **Percentage trimmed** | **Avg. Length after trim** |
| --- | --- | --- | --- | --- | --- | --- |
| NYCIII | M1 | 13507629 | 89 | 13507626 | 100 | 88.82 |
|  | M2 | 18968645 | 89 | 18968645 | 100 | 88.82 |
|  | M3 | 20716702 | 89 | 20716698 | 100 | 88.80 |
| mGTS | M1 | 14929621 | 89 | 14929621 | 100 | 88.82 |
|  | M2 | 14307034 | 89 | 14307034 | 100 | 88.80 |
|  | M3 | 13137576 | 89 | 13137570 | 100 | 88.82 |

**Supplementary Table 4.** Reads mapping summary statistics for *G. vaginalis*, *F. vaginae*, and *P. bivia* in triple-species biofilms formed in both NYCIII and mGTS. The results represent the average for the three analyzed replicates

|  | | **Reads mapped** | | | **Reads not mapped** | | | **Total** | | |
| --- | --- | --- | --- | --- | --- | --- | --- | --- | --- | --- |
| **Condition** | **Species** | **Average Nr sequences** | **SD** | **%** | **Average Nr sequences** | **SD** | **%** | **Average Nr sequences** | **SD** | **%** |
| NYCIII | *G. vaginalis* | 1738180.33 | 944937.31 | 9.80 | 15992809.33 | 2816433.46 | 90.20 | 17730989.67 | 3760521.62 | 100.00 |
|  | *F. vaginae* | 13433645.33 | 2080592.37 | 75.76 | 4297344.33 | 1680029.00 | 24.24 | 17730989.67 | 3760521.62 | 100.00 |
|  | *P. bivia* (contig 1)* | 1017553.67 | 212958.04 | 5.74 | 16713436.00 | 3565808.03 | 94.26 | 17730989.67 | 3760521.62 | 100.00 |
|  | *P. bivia* (contig 2)* | 322683.67 | 76964.77 | 1.82 | 17408306.00 | 3683717.48 | 98.18 | 17730989.67 | 3760521.62 | 100.00 |
|  | *P. bivia* (contig 3)* | 2034453.67 | 487665.46 | 11.47 | 15696536.00 | 3283018.80 | 88.53 | 17730989.67 | 3760521.62 | 100.00 |
| mGTS | *G. vaginalis* | 9530945.67 | 1243044.09 | 67.48 | 4593796.00 | 958433.31 | 32.52 | 14124741.67 | 909826.67 | 100.00 |
|  | *F. vaginae* | 3180363.33 | 446969.16 | 22.52 | 10944378.33 | 1140653.50 | 77.48 | 14124741.67 | 909826.67 | 100.00 |
|  | *P. bivia* (contig 1)* | 1190990.33 | 426450.55 | 8.43 | 12933751.33 | 651006.52 | 91.57 | 14124741.67 | 909826.67 | 100.00 |
|  | *P. bivia* (contig 2)* | 88318.00 | 57304.78 | 0.63 | 14036423.67 | 888210.96 | 99.37 | 14124741.67 | 909826.67 | 100.00 |
|  | *P. bivia* (contig 3)* | 1471713.67 | 635700.38 | 10.42 | 12653028.00 | 665064.65 | 89.58 | 14124626.33 | 909673.63 | 100.00 |

**P. bivia* mapping sequence is divided into 3 contigs.


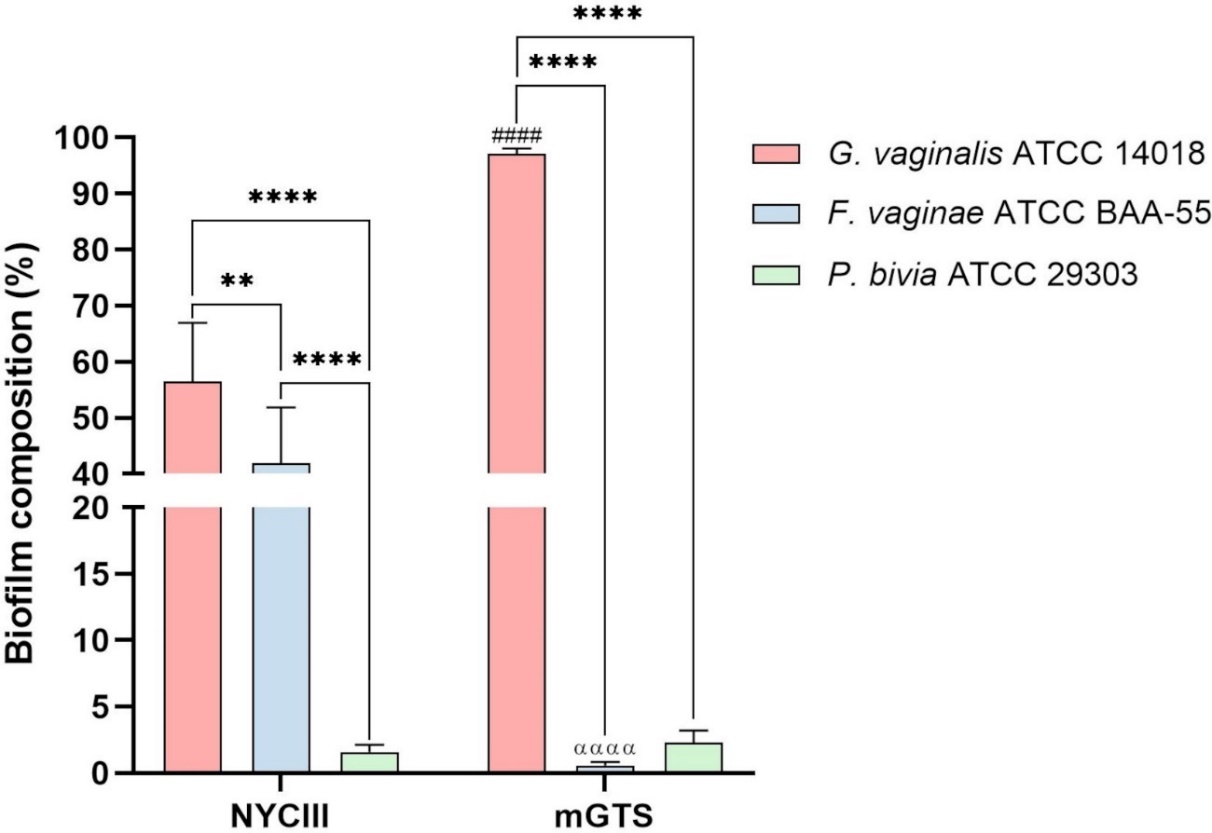


**Supplementary Figure 1.** Composition of triple-species biofilms formed in NYCIII and mGTS determined by qPCR. Results represent the average of each species percentage in the biofilm and error bars represent standard deviation. The results of biofilm composition in NYCIII were previously published (Sousa et al., 2024). Statistical analysis was performed using the two-way ANOVA with Tukey’s multiple comparisons test. Statistical significance between species in the same biofilm is represented by * (** *p* < 0.01; **** *p* < 0.0001). Significant differences between *G. vaginalis* in NYCIII and mGTS are represented by #### (*p* < 0.0001) and between *F. vaginae* in NYCIII and mGTS are represented by αααα (*p* < 0.0001). The graphic was plotted using GraphPad Prism. Comparing the biofilm composition in mGTS with the previous results of the composition of the biofilms in NYCIII (Sousa et al., 2024), in mGTS we observed an increase in the percentage of *G. vaginalis* and a slight increase in the percentage of *P. bivia*, however a significant decrease in the percentage of *F. vaginae*. The very low amounts of *F. vaginae* in the biofilms formed in mGTS are likely the result of the lack of capacity of this species to survive in this media.


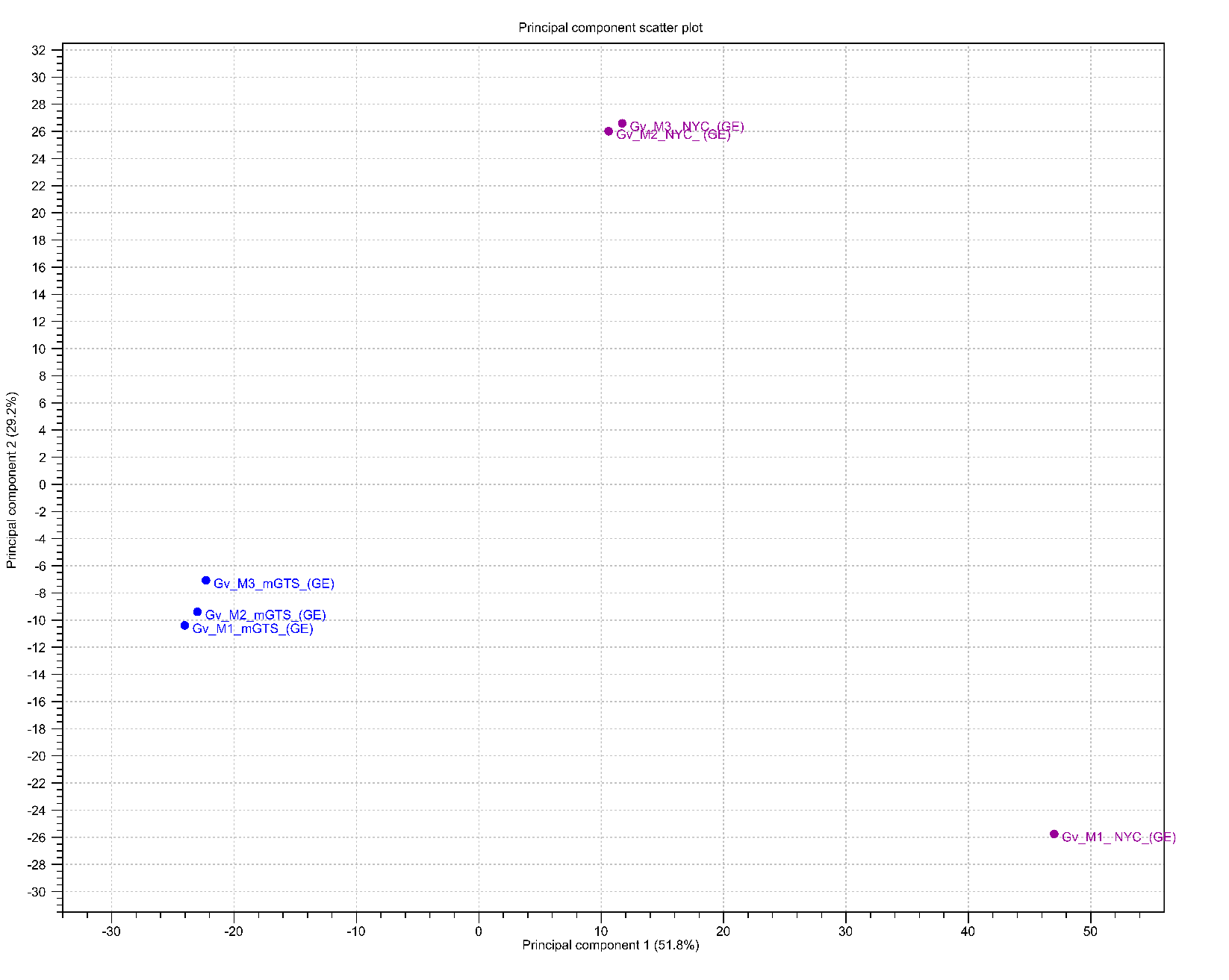
**Supplementary Figure 2.** Principal component analysis plot for *Gardnerella vaginalis*. The pink points represent the triplicates of triple-species biofilms grown in the New York City III medium (NYC) and the blue represent the triplicates of triple-species biofilms grown in the medium simulating genital tract secretions (mGTS). The PCA was plotted using the CLC genomics software.


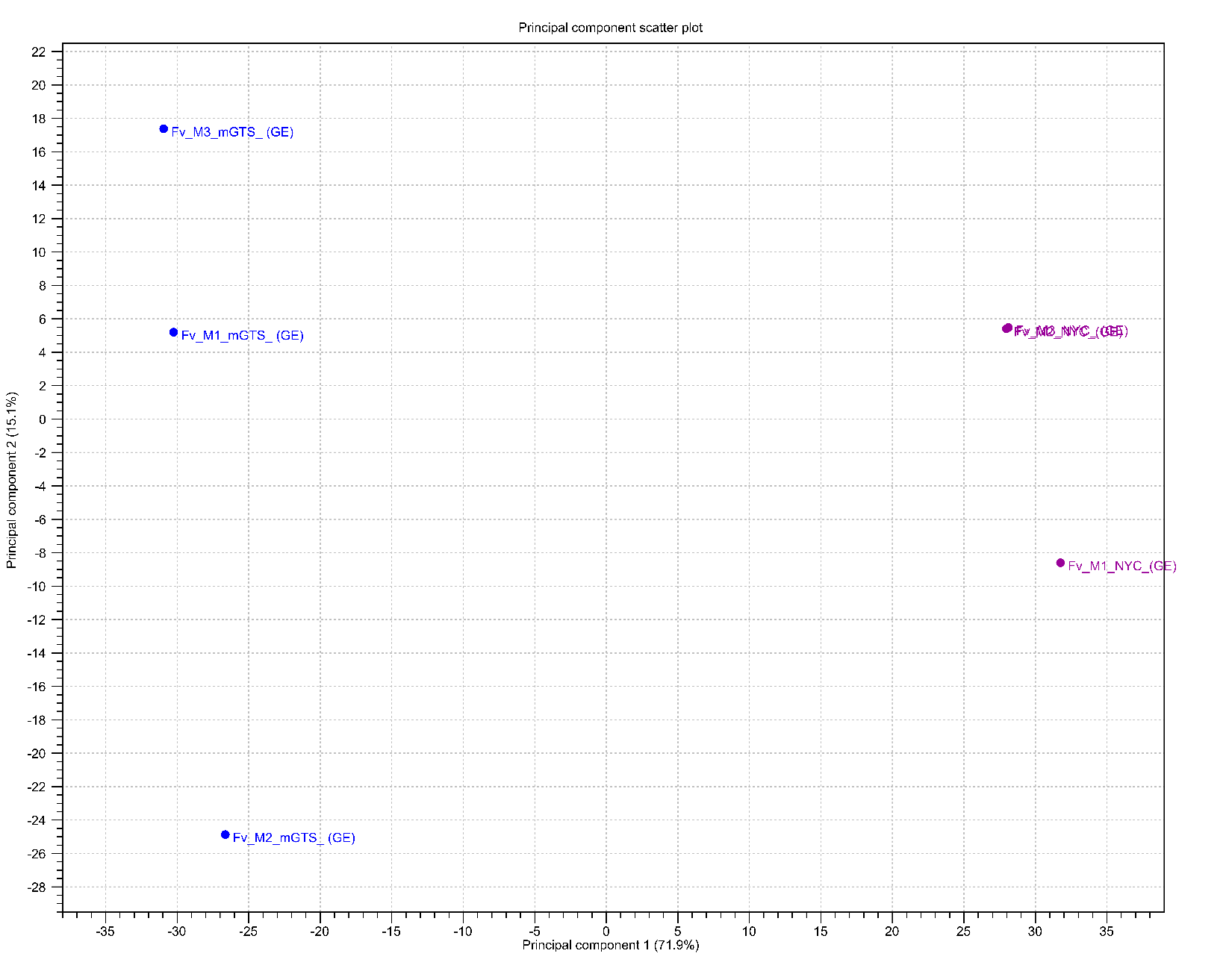
**Supplementary Figure 3.** Principal component analysis plot for *Fannyhessea vaginae*. The pink points represent the triplicates of triple-species biofilms grown in the New York City III medium (NYC) and the blue represent the triplicates of triple-species biofilms grown in the medium simulating genital tract secretions (mGTS). The PCA was plotted using the CLC genomics software.

**Supplementary Figure 4.** Principal component analysis plot for scaffold 1 of *Prevotella bivia*. The pink points represent the triplicates of triple-species biofilms grown in the New York City III medium (NYC) and the blue represent the triplicates of triple-species biofilms grown in the medium simulating genital tract secretions (mGTS). The PCA was plotted using the CLC genomics software.


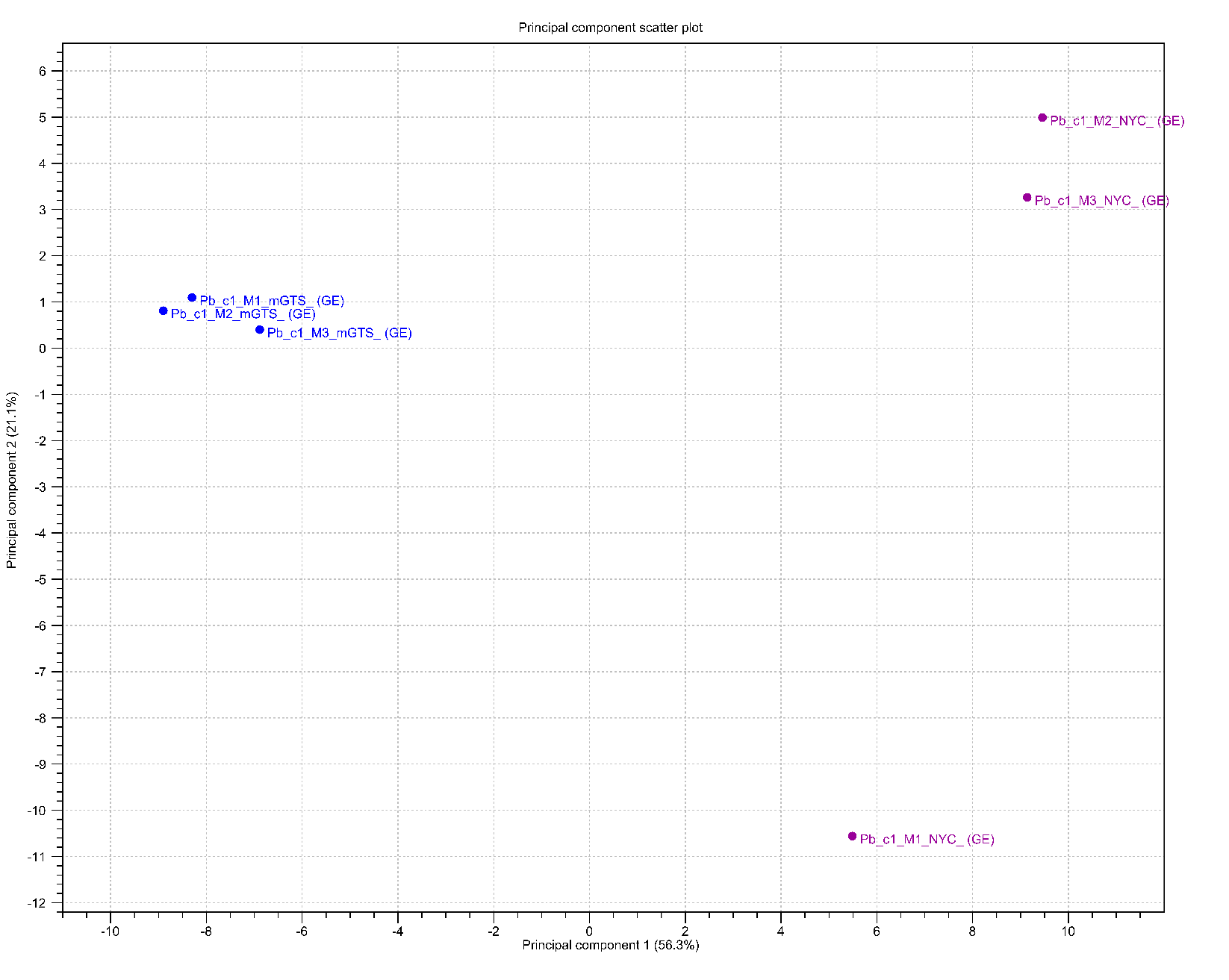


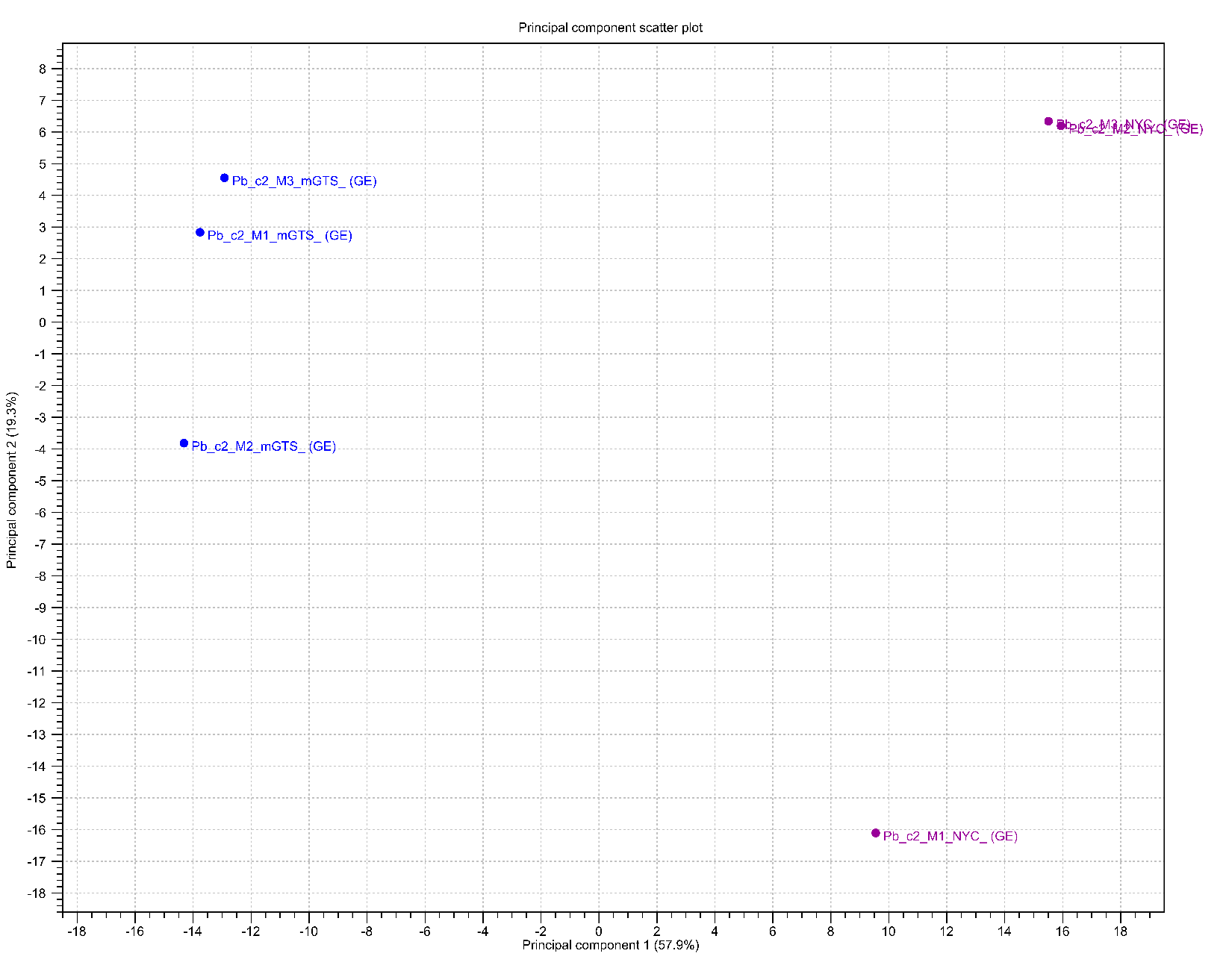
**Supplementary Figure 5.** Principal component analysis plot for scaffold 2 of *Prevotella bivia*. The pink points represent the triplicates of triple-species biofilms grown in the New York City III medium (NYC) and the blue represent the triplicates of triple-species biofilms grown in the medium simulating genital tract secretions (mGTS). The PCA was plotted using the CLC genomics software.


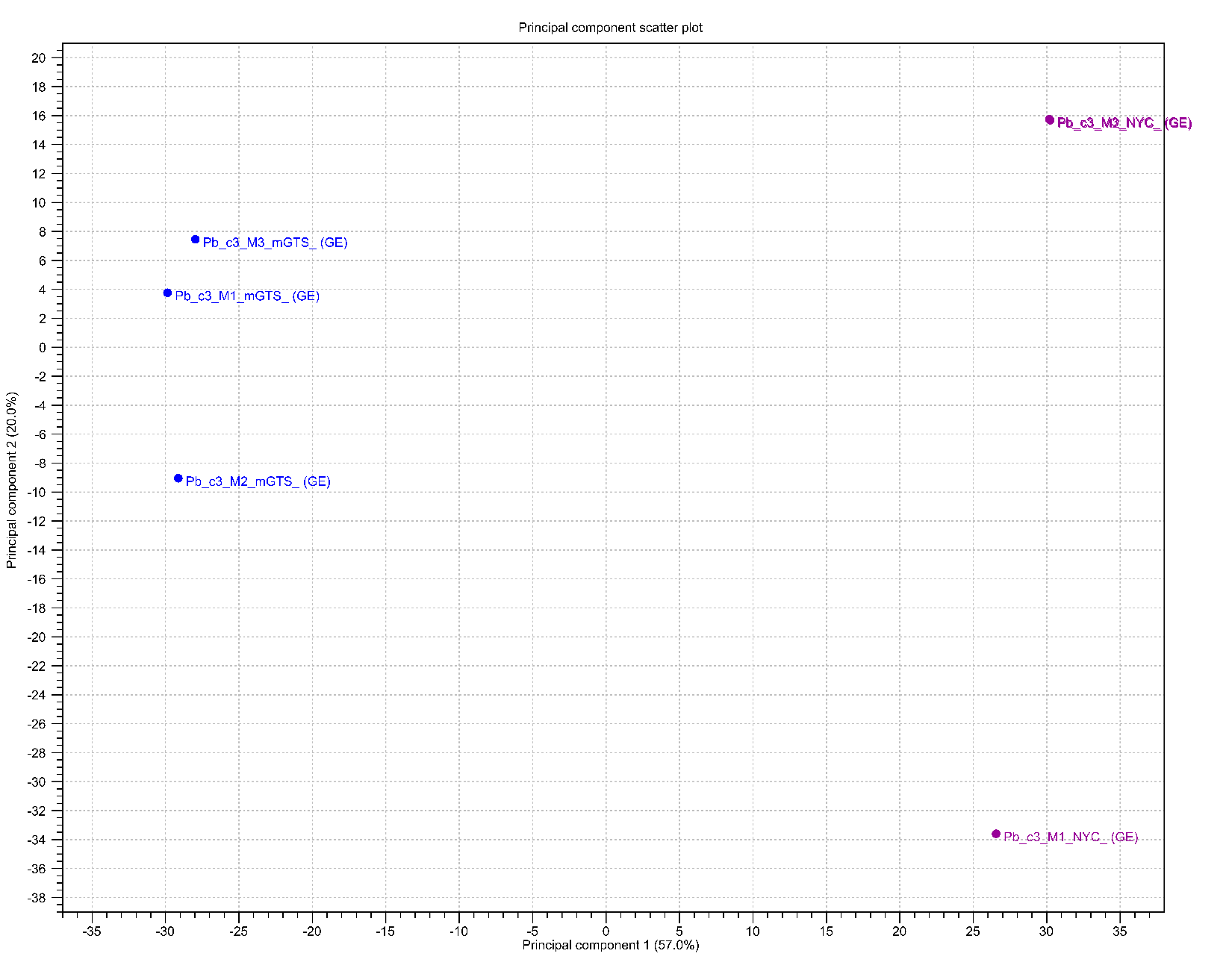
**Supplementary Figure 6.** Principal component analysis plot for scaffold 3 of *Prevotella bivia*. The pink points represent the triplicates of triple-species biofilms grown in the New York City III medium (NYC) and the blue represent the triplicates of triple-species biofilms grown in the medium simulating genital tract secretions (mGTS). The PCA was plotted using the CLC genomics software.


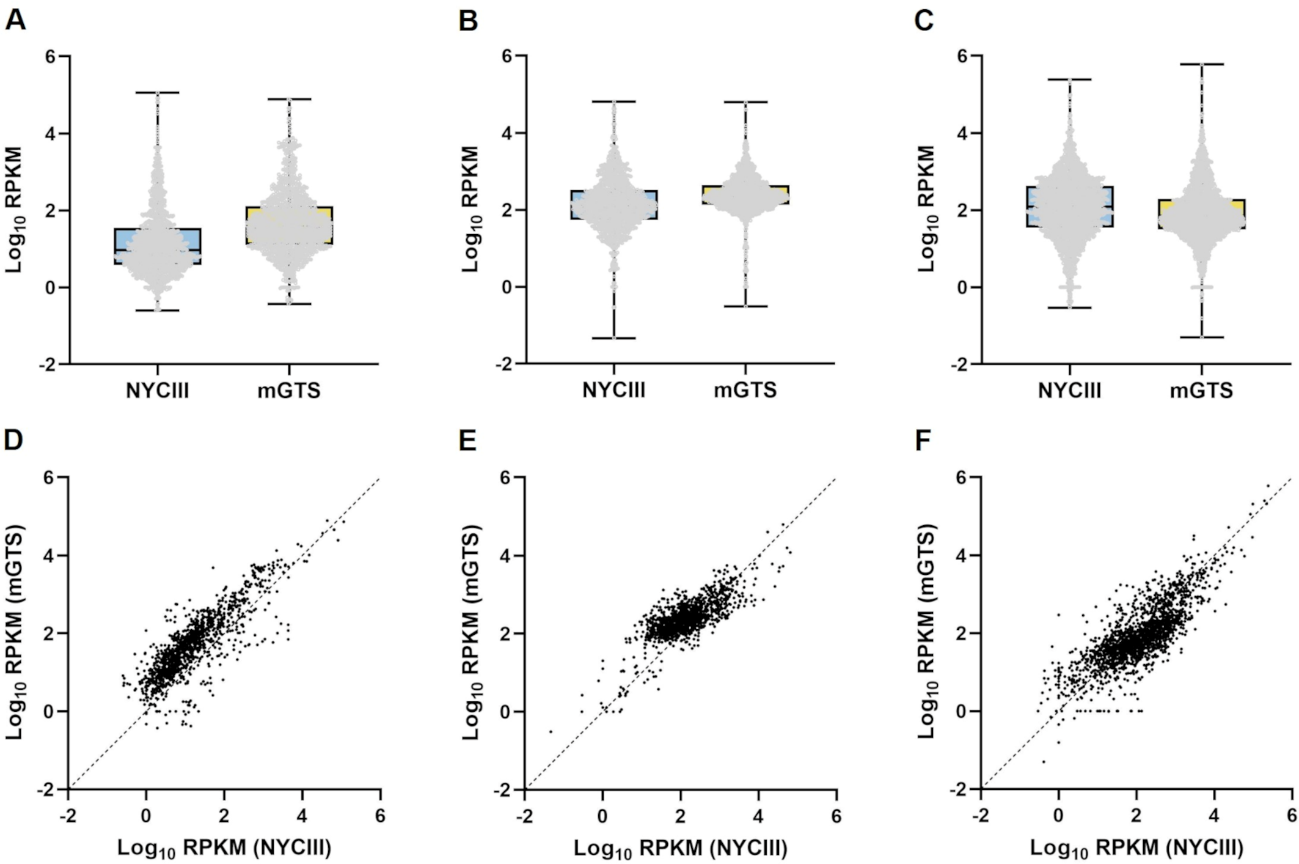


**Supplementary Figure 7.** Density distribution of RPKM values among conditions. Distribution of RPKM values for triple-species biofilms formed in the New York City III (NYCIII) medium and the medium simulating genital tract secretions (mGTS) for *Gardnerella vaginalis* (**A**), *Fannyhessea vaginae* (**B**), and *Prevotella bivia* (**C**). Correlation between RPKM values on the NYCIII and mGTS conditions for *G. vaginalis* (**D**), *F. vaginae* (**E**), and *P. bivia* (**F**). Graphics were plotted using GraphPad Prism.

**Supplementary Figure 8.** Heatmap showing gene expression in *Gardnerella vaginalis*. The heatmap was plotted using the CLC genomics software, with Eucledian distance as the clustering metric. Triplicates of triple-species biofilms formed in the New York City III medium and medium simulating genital tract secretions are represented by NYC and mGTS, respectively. The color scale indicates log counts per million, from lower (blue) to higher (red) gene expression levels.


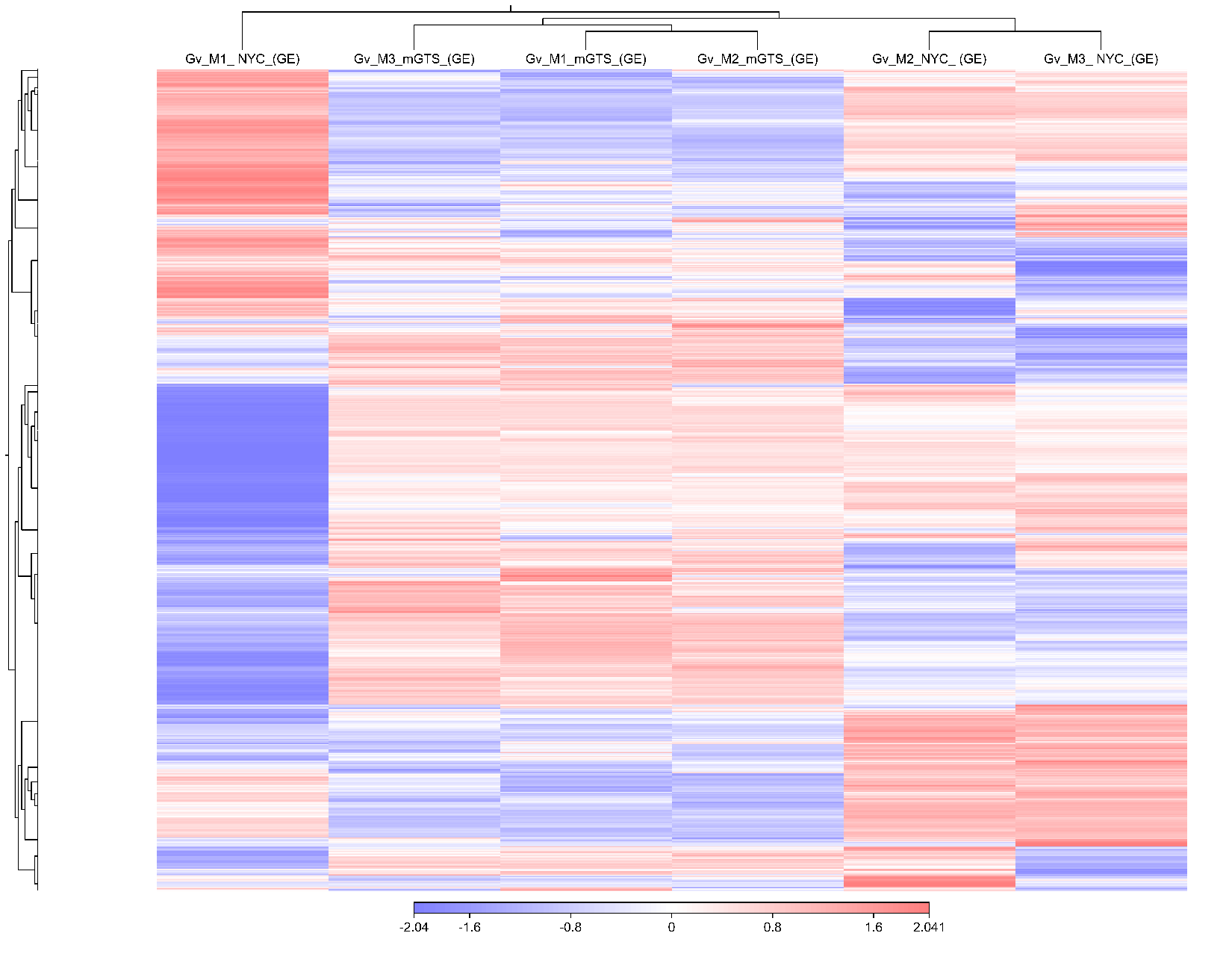


**Supplementary Figure 9.** Heatmap showing gene expression in *Fannyhessea vaginae*. The heatmap was plotted using the CLC genomics software, with Eucledian distance as the clustering metric. Triplicates of triple-species biofilms formed in New York City III medium and medium simulating genital tract secretions are represented by NYC and mGTS, respectively.
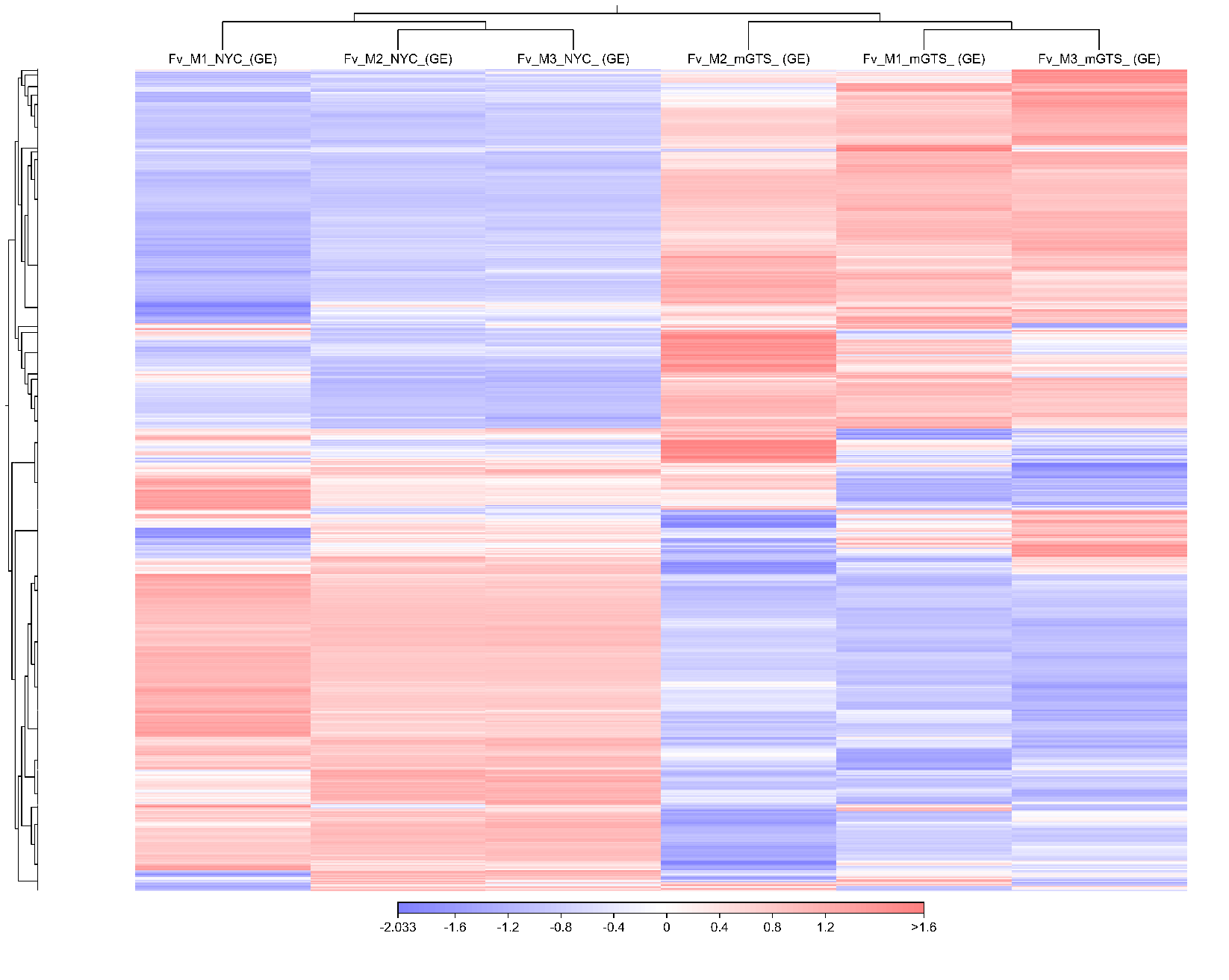
 The color scale indicates log counts per million, from lower (blue) to higher (red) gene expression levels.


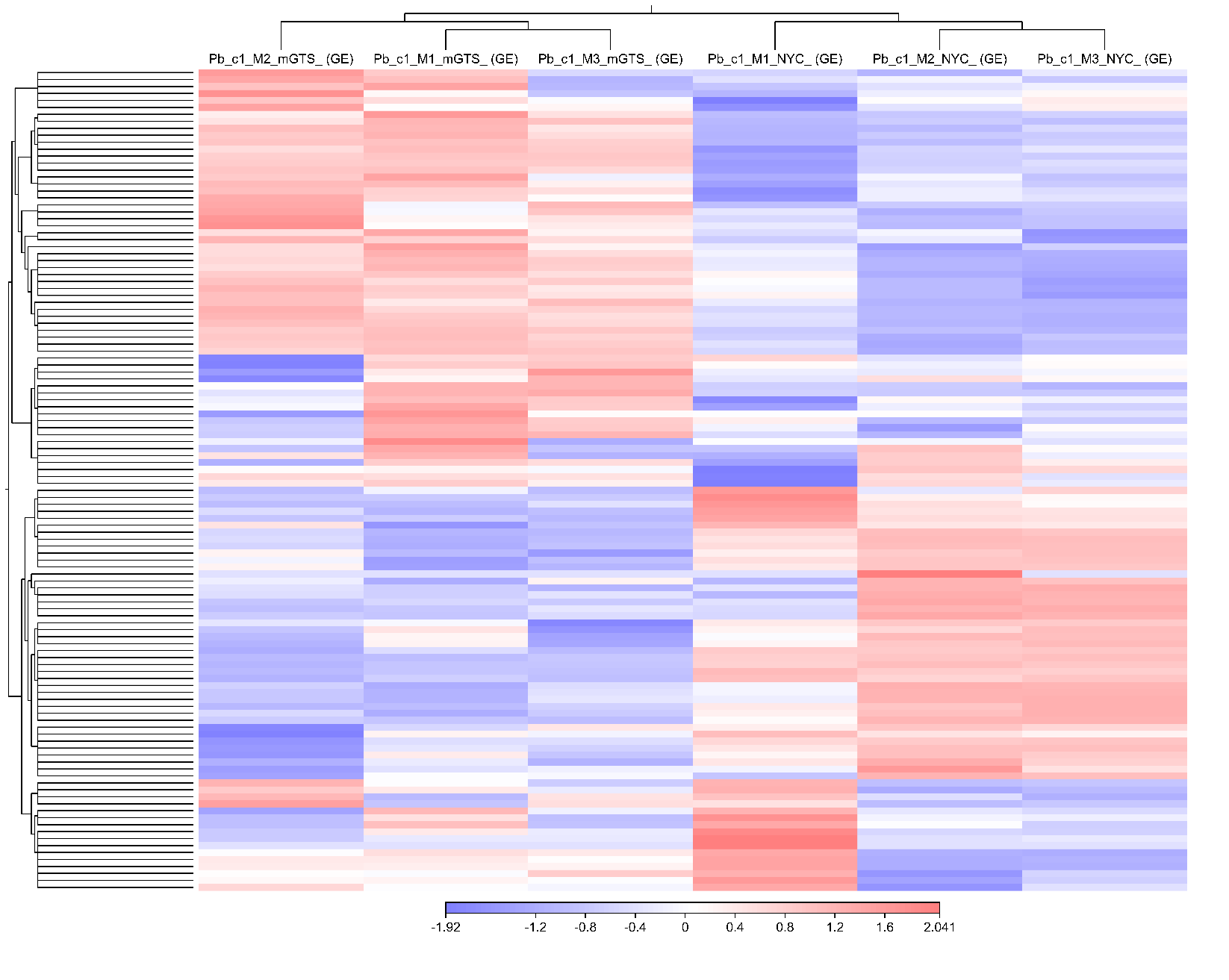
**Supplementary Figure 10.** Heatmap showing gene expression on scaffold 1 of *Prevotella bivia*. The heatmap was plotted using the CLC genomics software, with Eucledian distance as the clustering metric. Triplicates of triple-species biofilms formed in New York City III medium and medium simulating genital tract secretions are represented by NYC and mGTS, respectively. The color scale indicates log counts per million, from lower (blue) to higher (red) gene expression levels.


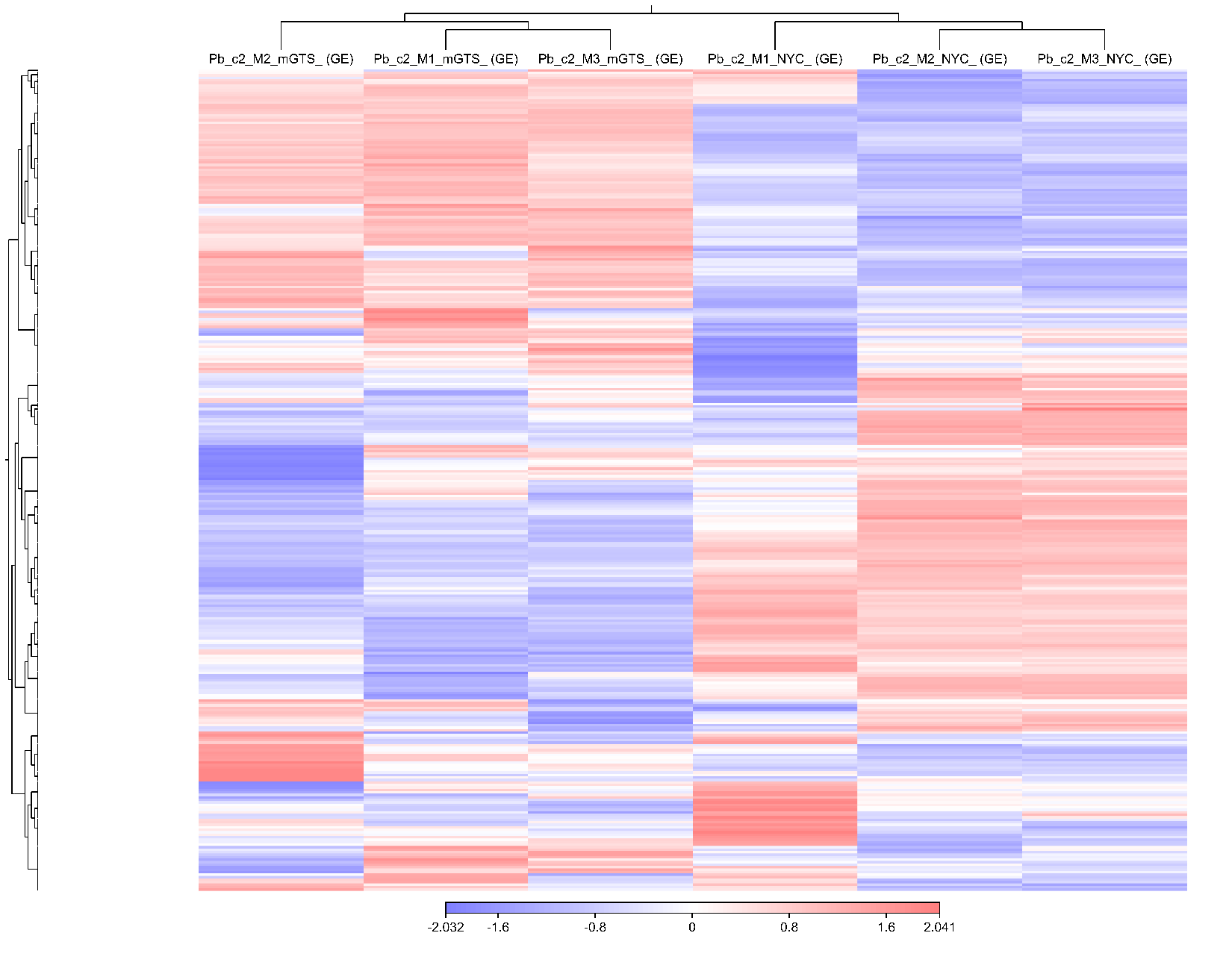
**Supplementary Figure 11.** Heatmap showing gene expression on scaffold 2 of *Prevotella bivia*. The heatmap was plotted using the CLC genomics software, with Eucledian distance as the clustering metric. Triplicates of triple-species biofilms formed in New York City III medium and medium simulating genital tract secretions are represented by NYC and mGTS, respectively. The color scale indicates log counts per million, from lower (blue) to higher (red) gene expression levels.


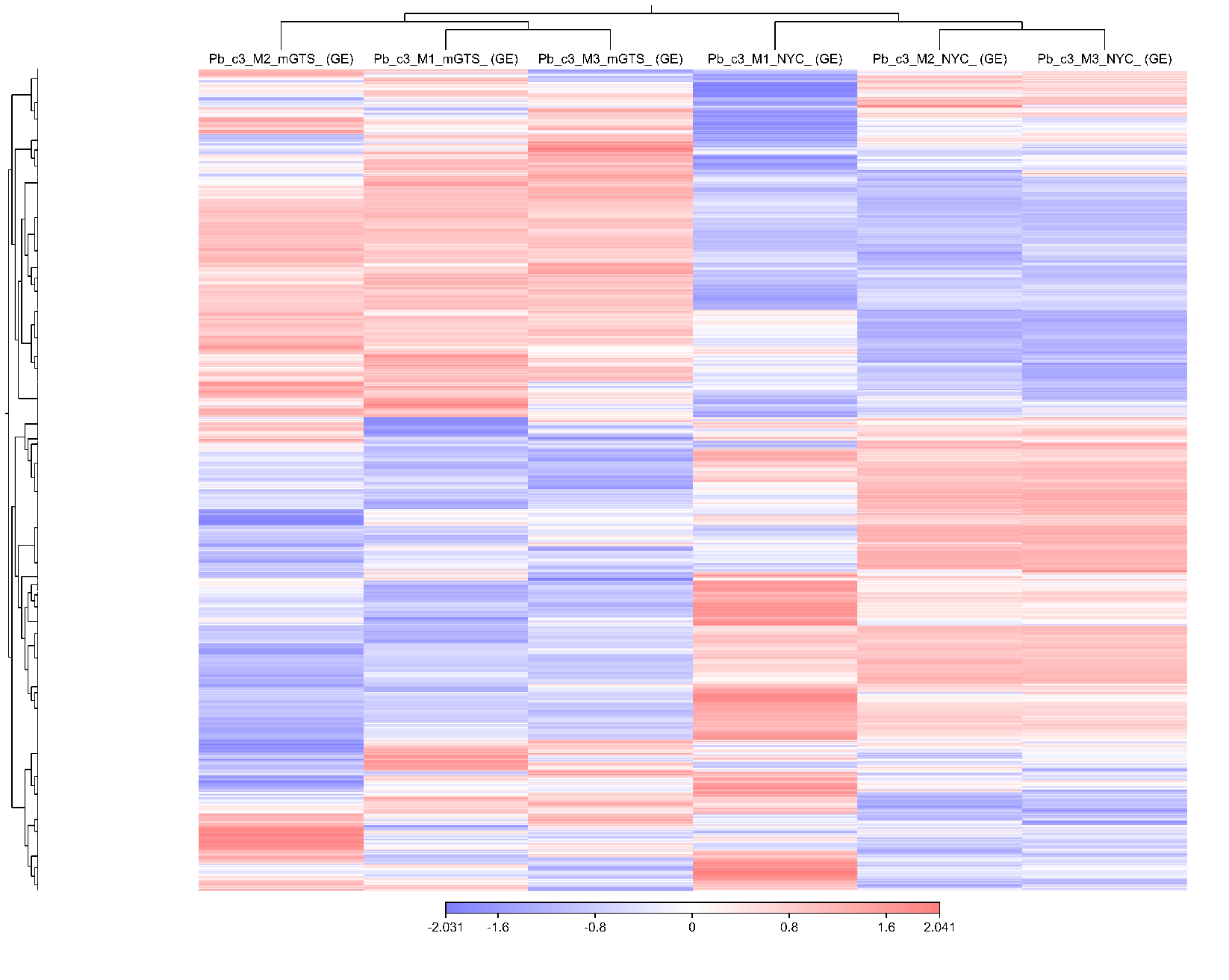
**Supplementary Figure 12.** Heatmap showing gene expression on scaffold 3 of *Prevotella bivia*. The heatmap was plotted using the CLC genomics software, with Eucledian distance as the clustering metric. Triplicates of triple-species biofilms formed in New York City III medium and medium simulating genital tract secretions are represented by NYC and mGTS, respectively. The color scale indicates log counts per million, from lower (blue) to higher (red) gene expression levels.

**
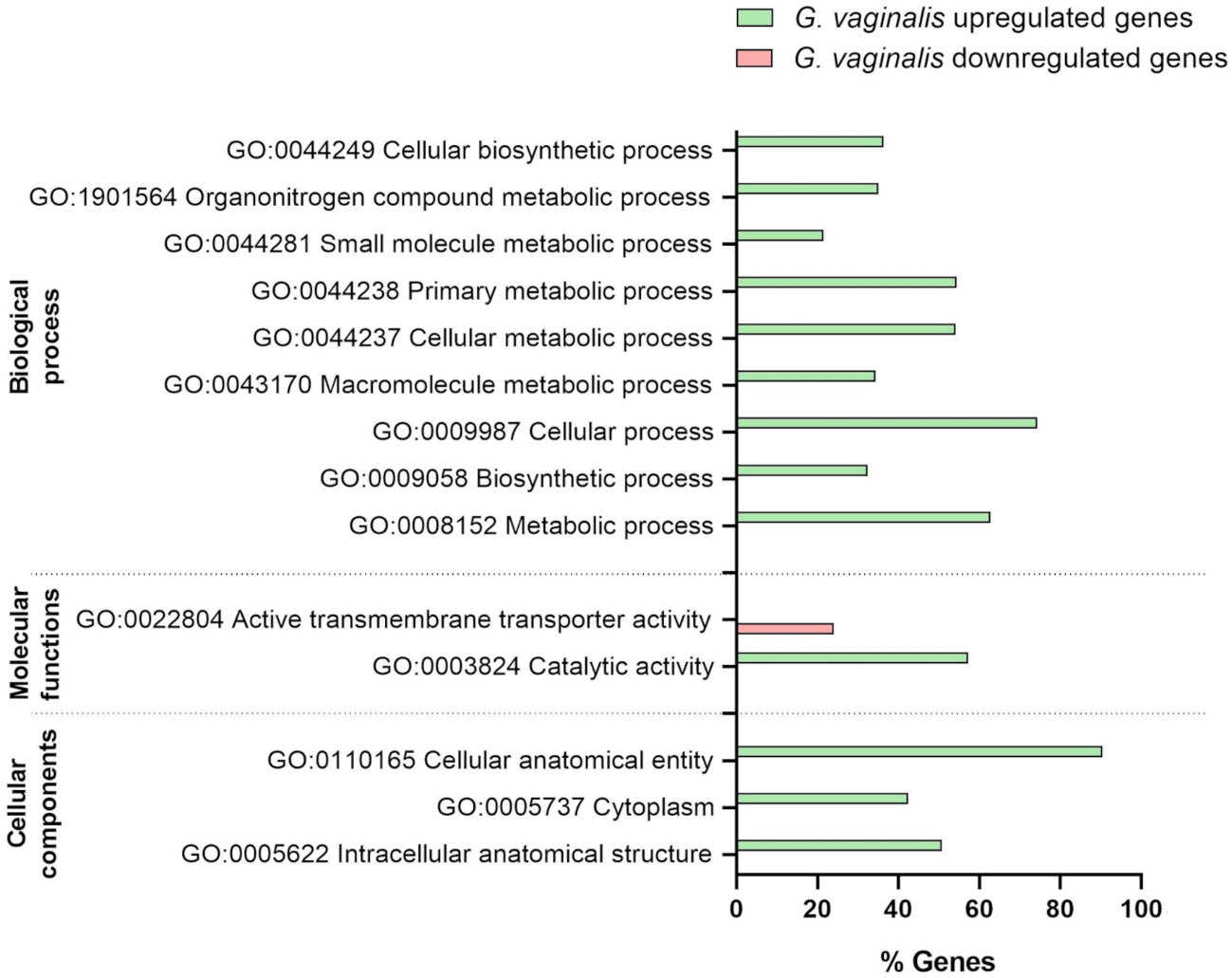
Supplementary Figure 13.** Gene ontology terms found enriched within *Gardnerella vaginalis* differentially expressed genes (FDR-adjusted *p*-value < 0.05). The figure was created using GraphPad Prism.

**
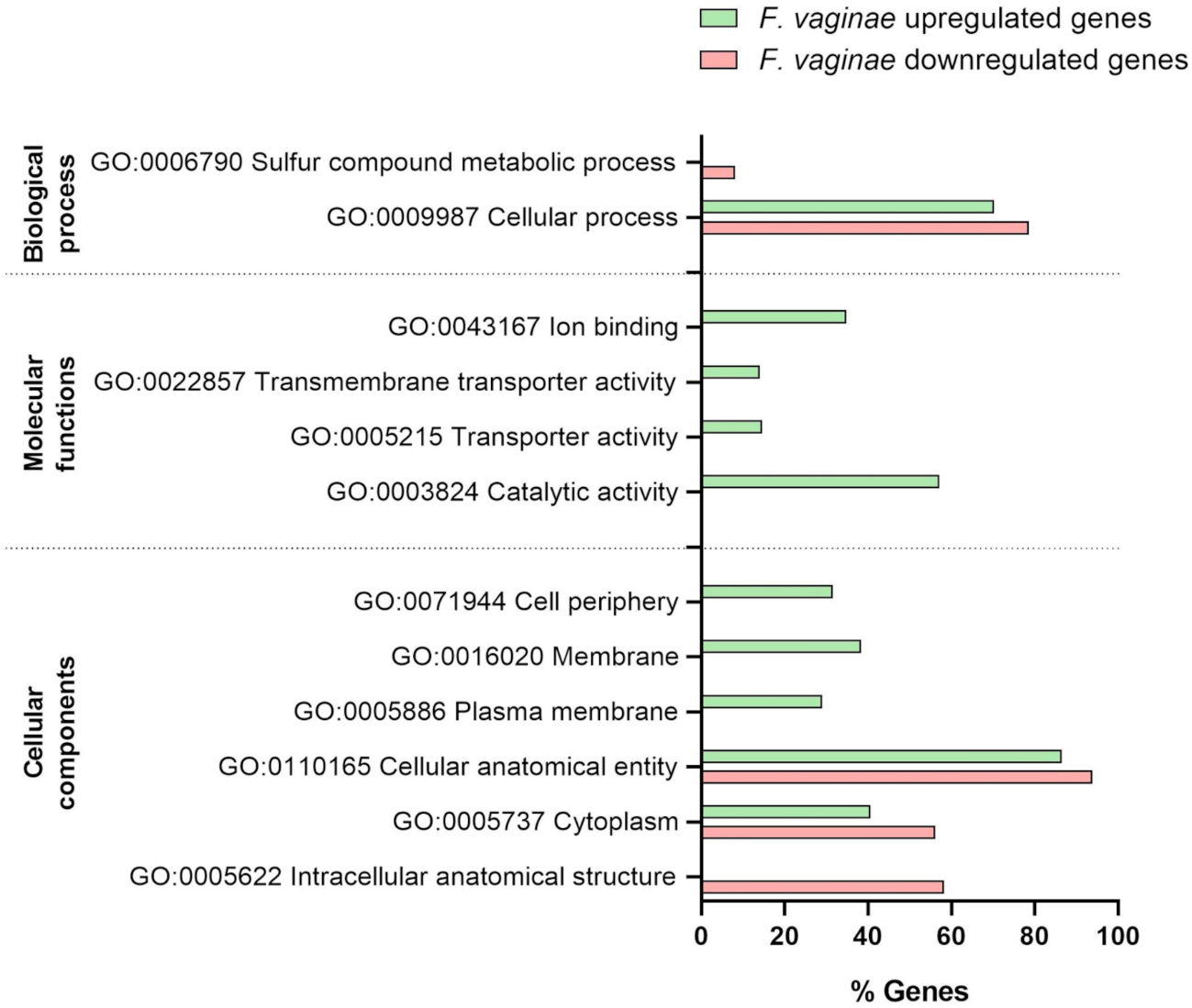
**

**Supplementary Figure 14.** Gene ontology terms found enriched within *Fannyhessea vaginae* differentially expressed genes (FDR-adjusted *p*-value < 0.05). The figure was created using GraphPad Prism.


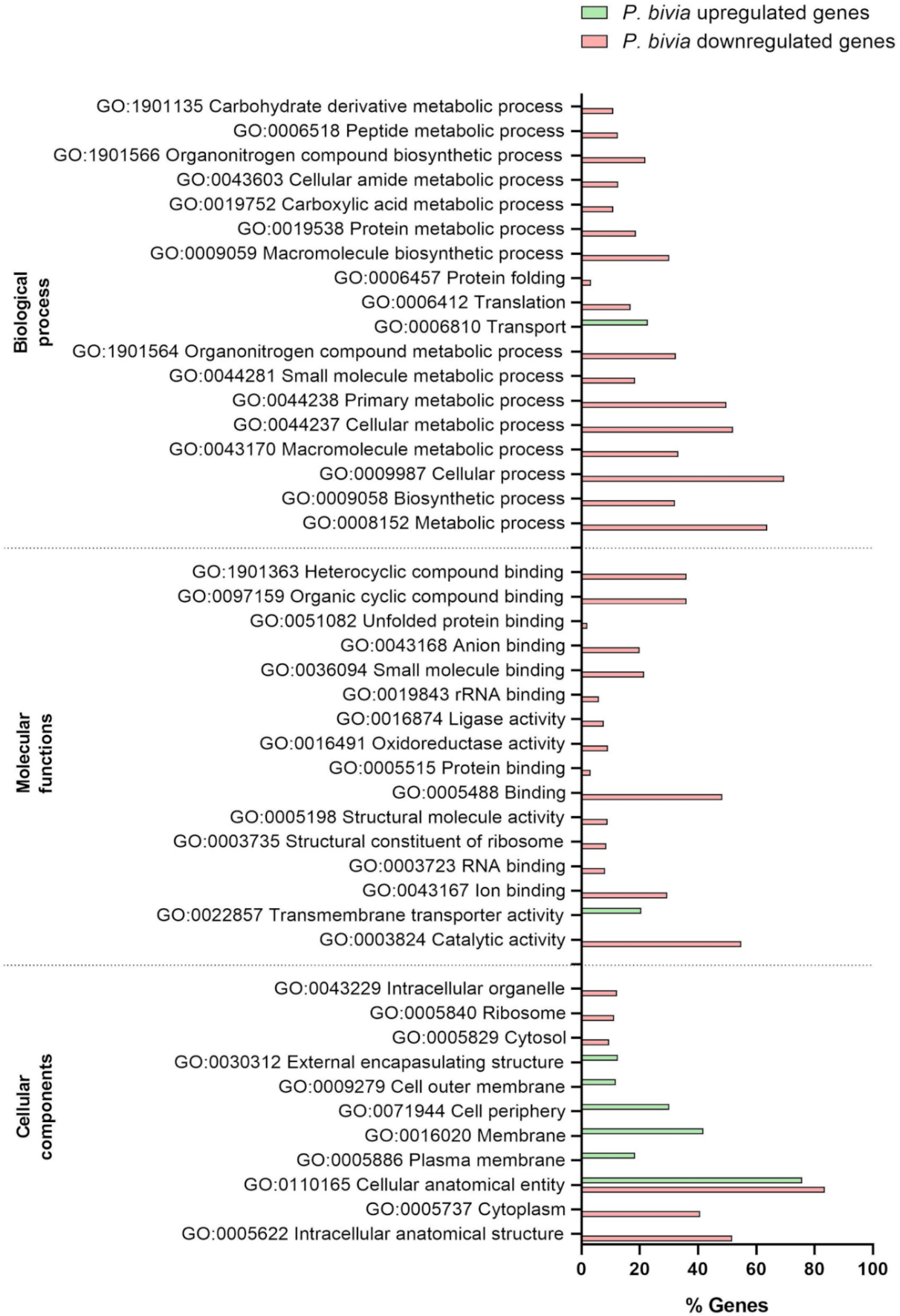
**Supplementary Figure 15.** Gene ontology terms found enriched within *Prevotella bivia* differentially expressed genes (FDR-adjusted *p*-value < 0.05). The figure was created using GraphPad Prism.


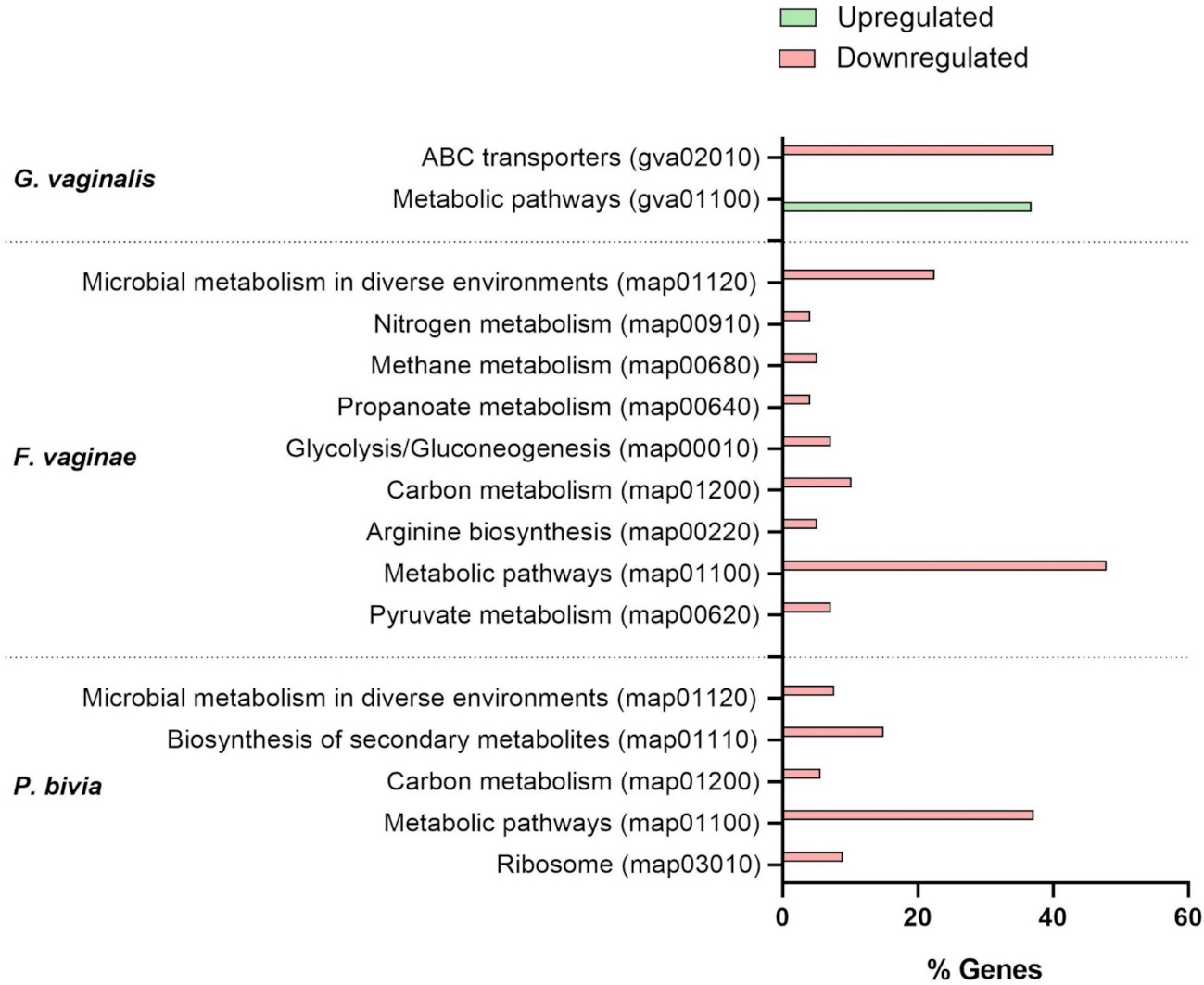
**Supplementary Figure 16:** Kyoto Encyclopedia of Genes and Genomes pathways found enriched within *Gardnerella vaginalis*, *Fannyhessea vaginae*, and *Prevotella bivia* differentially expressed genes (FDR-adjusted *p*-value < 0.05). The figure was created using GraphPad Prism.
